# Safety, Feasibility of Intravenous and Intrathecal Injection of Autologous Bone Marrow Derived Mesenchymal Stromal Cells in Patients with Amyotrophic Lateral Sclerosis: An Open Label Phase I Clinical Trial

**DOI:** 10.22074/cellj.2019.5370

**Published:** 2018-08-07

**Authors:** Seyed Massood Nabavi, Leila Arab, Neda Jarooghi, Tina Bolurieh, Fatemeh Abbasi, Soura Mardpour, Vajihe Azimyian, Fatemeh Moeininia, Saman Maroufizadeh, Leila Sanjari, Seyedeh Esmat Hosseini, Nasser Aghdami

**Affiliations:** 1 Department of Regenerative Medicine, Cell Science Research Center, Royan Institute for Stem Cell Biology and Technology, ACECR, Tehran, Iran; 2 Department of Neuroscience, School of Advanced Technologies in Medicine, Tehran University of Medical Science, Tehran, Iran; 3 Department of Epidemiology and Reproductive Health, Reproductive Epidemiology Research Center, Royan Institute for Reproductive Medicine, ACECR, Tehran, Iran; 4 Intensive Care Unit, Mostafa Khomeini Hospital, Tehran, Iran; 5 Student Research Committee, School of Nursing and Midwifery, Shahid Beheshti University of Medical Sciences, Tehran, Iran

**Keywords:** Amyotrophic Lateral Sclerosis, Bone Marrow, Intrathecal, Intravenous, Mesenchymal Stromal Cell

## Abstract

**Objective:**

Amyotrophic lateral sclerosis (ALS) is the most severe disorder within the spectrum of motor neuron diseases (MND) that has no effective treatment and a progressively fatal outcome. We have conducted two clinical trials to assess the safety and feasibility of intravenous (IV) and intrathecal (IT) injections of bone marrow derived mesenchymal stromal cells (BM-MSCs) in patients with ALS.

**Materials and Methods:**

This is an interventional/experimental study. We enrolled 14 patients that met the following inclusion criteria: definitive diagnosis of sporadic ALS, ALS Functional Rating Scale (ALS-FRS) ≥24, and ≥40% predicted forced vital capacity (FVC). All patients underwent bone marrow (BM) aspiration to obtain an adequate sample for cell isolation and culture. Patients in group 1 (n=6) received an IV and patients in group 2 (n=8) received an IT injection of the cell suspension. All patients in both groups were followed at 24 hours and 2, 4, 6, and 12 months after the injection with ALS-FRS, FVC, laboratory tests, check list of side effects and brain/spinal cord magnetic resonance imaging (MRI). In each group, one patient was lost to follow up one month after cell injection and one patient from IV group died due to severe respiratory insufficiency and infection.

**Results:**

During the follow up there were no reports of adverse events in terms of clinical and laboratory assessments. In MRI, there was not any new abnormal finding. The ALS-FRS score and FVC percentage significantly reduced in all patients from both groups.

**Conclusion:**

This study has shown that IV and IT transplantation of BM-derived stromal cells is safe and feasible (Registration numbers: NCT01759797 and NCT01771640).

## Introduction

Amyotrophic lateral sclerosis (ALS) is one of the most damaging motor neuron diseases (MNDs) that has a worldwide incidence of 2-3 per 100,000 (1). Until now, there is no effective medication to halt disease progression or provide a cure. Available treatments are limited to pharmaceuticals (riluzole) (2), physical and speech therapy (3), nutrition, and respiratory support (4, 5). In the last decade, stem cell transplantation has been considered as a promising therapeutic option for these patients (6). Recent studies demonstrated the safety and efficacy of different types of stem cell transplantations in ALS patients such as peripheral blood stem cells (PBSC) (7, 8), mesenchymal stromal cells (MSCs) (9-15), olfactory ensheathing cells (OEC) (16) and fetal neural stem cells (NSC) (17-19). One of the most considerable stem cells are MSCs which use several mechanisms to correct ALS impairments such as rich trophic factor secretion, immunomodulation by increased expressions of interlukin-10 (IL-10) and Transforming growth factor beta-1 (TGF-ß1) (20), gene delivery or replacing lost cells (21). Therefore, MSCs could induce neuroprotective effects on glutamate excitoxicity by inhibiting the expression of N-methyl-D-aspartate (NMDA) receptor and controlling glutamate related Ca^2+^ influx (22). 

GABAergic transmission increases in neurons co- cultured with MSCs and can induce neural repair (23). Therefore, MSCs have the potential to improve neural function in a damaged area of the central nervous system (24-26). In an animal model of ALS, it has been shown that MSC transplantation in SOD1/G93A mice restored motor neurons, prolonged life span, and improved motor function by the secretion of growth factors, immunomodulatory effects, and reductions of oxidative stress (26) . In previous studies, stem cell transplantation was performed via different routes in ALS patients such as intrathecal (IT) (9, 20), intraspinal (27, 28), intravenous (IV) (7, 9), intraventricular (11), intracortical (29), and intra-arterial (30) injections. However, the preferred route of administration has yet to be determined in ALS. Therefore, we initiated this study to evaluate the safety of IV and IT injections of MSCs in ALS patients. As a secondary objective, we compared the effects of each route of injection on prevention of disease progression. 

## Materials and Methods

**Fig.1:**
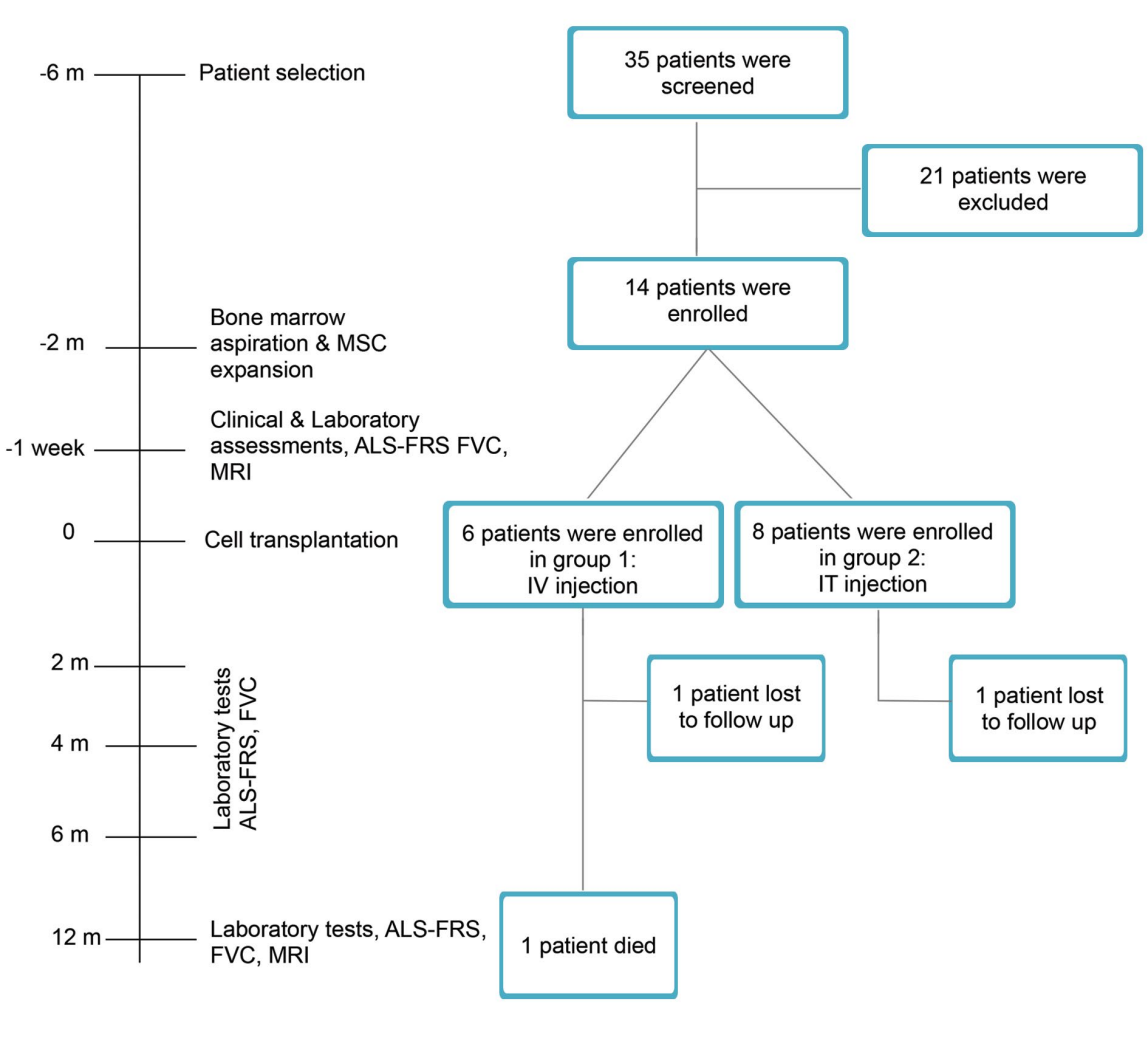
Study flow diagram.

This is an interventional/experimental study. We conducted these two clinical trials as phase 1 open label clinical studies at Royan Institute in collaboration with the Neurology Department of Mostafa Khomeini Hospital. After study approval from the Royan Institute Ethics Research Committee (No. EC/91/1097), eligible patients signed the informed consent and enrolled in the study. These studies were registered at the NIH clinical trial site (www.clinicaltrials.gov) with identification numbers NCT01759797 and NCT01771640. Figure 1 shows the study flowchart. 

### Patients

A total of 14 male and female patients with definitive diagnosis of sporadic ALS, aged 24-60 years, enrolled in the studies. All patients had definite diagnosis of ALS due to EL Escorial criteria (31) and more than 6 months of evolution of disease, an ALS Functional Rating Scale (ALS-FRS) score =24, and =40% predicted forced vital capacity (FVC). All patients were treated by the only approved drug for ALS, riluzole, at a dose of 100 mg, twice per day. Exclusion criteria were any concomitant neurological, psychiatric or systemic diseases or use of any corticosteroids, immunoglobulin, or immunosuppressant treatments during 6 months before enrollment. Patients’ descriptive characteristics are listed in Table 1.

### Bone marrow derived mesenchymal stromal cells production

Each patient underwent bone marrow aspiration from the posterior superior iliac crest while in the right or left lateral positions under local anesthesia. The MSCs were prepared from bone marrow sample (100 ml) according to current good manufacturing practice (cGMP).

Mononuclear cells (MNCs) were isolated from the BM samples by density gradient with a Ficoll Paque open system (Lymphodex, Inno-Train, Germany). Next, the MNC layer was isolated and washed in PBS buffer (Milteny Biotech GmbH, Germany). Cell counts and viability were assessed with trypan blue staining and confirmed by a NucleoCounter® system (ChemoMetec, Denmark). MNCs (1×10^6^/cm^2^) were placed in Millicell® HY T-600 culture flasks (Merk, Germany) and cultured under standard conditions in 1X MEM alpha medium (Gibco, Germany) and fetal bovine serum (FBS, Gibco, Germany). Flasks were incubated under defined conditions of 5% CO_2_ and 37°C. All non-adherent cells were removed by changing the culture medium after 3-4 days. This process was repeated every 3 days. After 1 to 2 passages, the 90% confluent MSCs were harvested by the application of 0.25% trypsin in 0.1% Ethylenediaminetetraacetic acid (EDTA). Cell viability was evaluated by trypan blue staining as well as the NucleoCounter® system. Next, we suspended MSCs (2×10^6^ cells/kg) in 5 ml of 0.9% sodium chloride that contained 2% human serum albumin.

### Flow cytometry

Cell surface marker expressions were assessed by flow cytometry. The characterization panel consisted of monoclonal antibodies for mesenchymal lineages markers CD90-FITC (BD, Pharmingen^TM^, USA), CD105PE (Endoglin, BD Pharmingen^TM^, USA), CD73-PE (BD Pharmingen^TM^, USA), CD44-FITC (BD Pharmingen^TM^, USA), CD45 FITC-CD34 PE (BD Pharmingen^TM^, USA) and CD11b (BD Pharmingen^TM^, USA), along with the following isotype controls, MultiMixTM FITC Mouse IgG1, PE-Mouse IgG1 (Dako, Denmark), FITC-Mouse IgG2b (Millipore, USA), and PE-conjugated Mouse IgG1k (BD Pharmingen^TM^, USA). Cells were fixed with 4% paraformaldehyde and immunophenotyping analysis was performed by the BD FACS Calibur flow cytometry system (BD Biosciences, USA).

### Cell transplantation

Patients were scheduled to receive either the IV or IT injections of the prepared MSC suspension (2×10^6^ cells/ kg) (Table 2). After cell transplantation, each patient remained under close observation for 24 hours. Then, we followed them with regular assessments at 24 hours, and 2, 4, 6, and 12 months after the cell administration.

**Table 1:** Patients’ characteristics


Patient	Age (Y)	Sex	ALS onset	ALS duration (months from diagnosis)	PEG (months from injection)	Tracheostomy (months from injection)

1	50	Male	Limb	18	8	10
2	59	Male	Bulbar	6	N/A	N/A
3	60	Female	Limb	18	N/A	N/A
4	35	Female	Limb	6	N/A	N/A
5	34	Female	Limb	10	N/A	N/A
6	57	Female	Limb	12	N/A	N/A
7	51	Male	Bulbar	6	N/A	N/A
8	54	Male	Bulbar	6	N/A	N/A
9	53	Male	Bulbar	30	N/A	N/A
10	57	Female	Limb	48	N/A	N/A
11	31	Male	Limb	12	N/A	N/A
12	42	Male	Limb	9	N/A	N/A
13	39	Male	Limb	24	4	4
14	24	Male	Limb	24	N/A	N/A


N/A; Not applicable, ALS; Amyotrophic lateral sclerosis, and PEG; Percutaneous endoscopic gastrostomy.

**Table 2:** Cell information


Patient	Route of injection	Cell count (×10^6^)	Cell viability (%)	Bacteriology	Mycoplasma	Endotoxin level (EU/ml)	Karyotype

1	IV	95	100	NC	NC	<0.125	46XY
2	IV	125	98	NC	NC	<0.125	46XY
3	IV	75	92.80	NC	NC	<0.125	46XX
4	IV	111	94	NC	NC	<0.125	46XX
5	IV	89	93	NC	NC	<0.125	46XX
7	IT	113	99.50	NC	NC	<0.125	46XY
8	IT	102	94	NC	NC	<0.125	46XY
9	IT	100	98	NC	NC	<0.125	46XY
10	IT	135	97	NC	NC	<0.125	46XX
11	IT	140	96	NC	NC	<0.125	46XY
12	IT	100	96	NC	NC	<0.125	46XY
13	IT	120	98	NC	NC	<0.125	46XY


IV; Intravenous, IT; Intrathecal, NC; No contamination, and EU; Endotoxin unit.

### Drug administration 

All patients took rilozule (100 mg) twice a day. If needed, patients received medications for symptom control or nursing support.

### Clinical assessment

The assessments included the comprehensive physical examination, taking history about any new symptoms, ALS-FRS (32, 33), FVC, laboratory analysis (liver, kidney, thyroid function, serology, virology, urine analysis and culture). We performed them at 6, 4, 2, and one week before cell therapy and also 2, 4, 6, and 12 months after cell transplantation.

### Magnetic resonance imaging

Brain and spinal cord MRIs were performed one week before and 12 months after the cell transplantation. The system used for scanning was the 1.5 Tesla system, (GE System version 2000). The images were taken in the sagittal, axial, and coronal planes with T1, T2 and flair fast spin-echo sequence.

### Statistical analysis

We carried out the statistical analysis with IBM SPSS Statistics for Windows, Version 22.0 (IBM Corp., Armonk, NY, USA). In the present study, continuous variables were expressed as mean ± standard error (SE). Repeated-measures ANOVA were used to assess the effects of treatment and time (months) and treatment-by-time interactions on ALS-FRS and FVC. All statistical tests were 2-sided and P<0.05 was considered statistically significant.

## Results

### Flow cytometry

**Fig.2:**
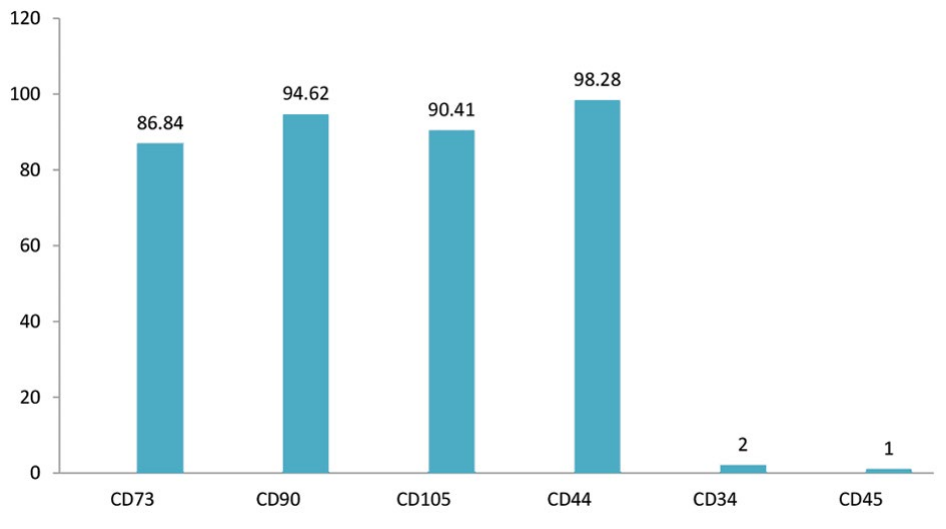
Expressions of bone marrow derived mesenchymal stromal cell (BM-MSC) surface markers.

Flow cytometry analysis showed that BM-MSCs highly expressed the CD73, CD90, CD105, and CD44 markers and almost lacked expressions of CD34, CD45 (Fig .2).

### Adverse events

Of 14 patients, 1 patient from each group was lost to follow up after cell injection and we continued the study with 12 patients, 5 in IV group and 7 in IT group. 1 patient had hypotension (85/60 mmHg) immediately after the IV injection of the cell suspension. After infusion of 1000 ml 0.9% sodium chloride, the blood pressure increased to 105/80 mmHg with stable vital signs. In addition, 2 patients had headaches and nausea after the IT injection of the cell suspension that lasted for 24 hours, which we attributed to the lumbar puncture. After hydration and use of nonsteroidal anti-inflammatory drugs (NSAIDS), their symptoms resolved. In addition, there was no report of any major adverse events or new abnormal findings in the brain and spinal MRI scans during 12 months after the cell transplantation (Table 3).

**Table 3:** Adverse effects after cell transplantation


Adverse effect	Patient (n)	Time of occurrence (weeks)	Outcome

Neurological adverse events			
Unconsciousness	0	0	
Dizziness	0	0	
Headache	2	1	Improved after treatment
Neck stiffness	0	0	
Nausea and vomiting	2	1	Improved after treatment
Hypotension	1	1	Improved after treatment
Motor dysfunction	0	0	
Sensory dysfunction	0	0	
Sphincter dysfunction	0	0	
Seizures	0	0	
Vertigo	0	0	
Visual impairment	0	0	
Allergic reactions			
Fever	0	0	
Apnea	0	0	
Dyspnea	0	0	
Anaphylaxis	0	0	
Urticaria	0	0	
Erythema	0	0	
Flashing	0	0	
Local adverse events			
Phlebitis	0	0	
Infection	0	0	
Hematoma	0	0	
Other adverse events			
Diarrhea	0	0	
Constipation	0	0	
Bronchitis	0	0	
Pneumonia	0	0	
Pulmonary emboli	0	0	
Respiratory failure	0	0	
Arrhythmia	0	0	


### Follow up

**Fig.3:**
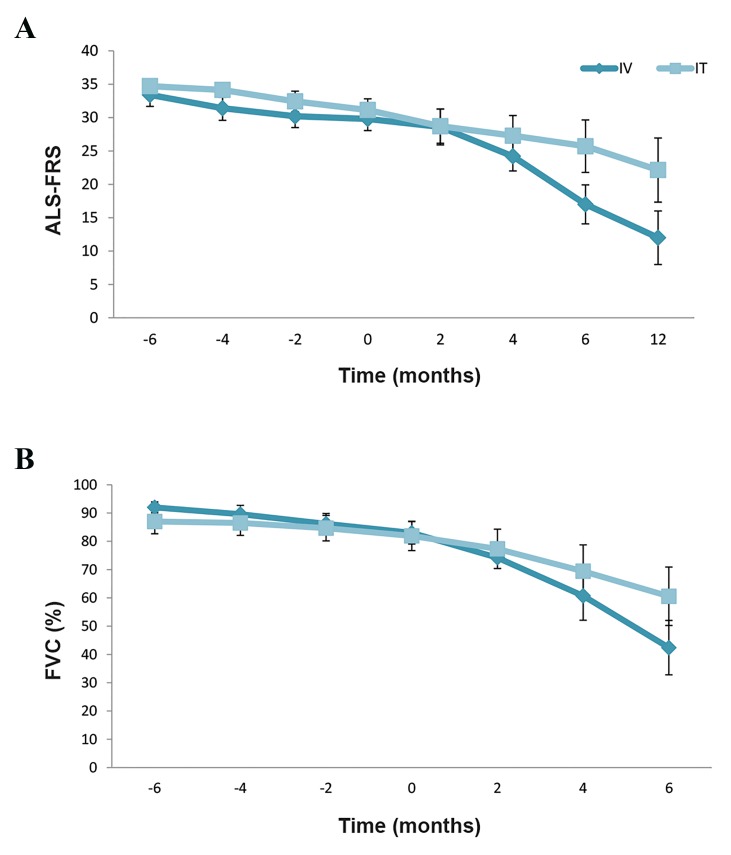
The trend of amyotrophic lateral sclerosis-functional rating scale (ALS-FRS) and forced vital capacity during 12 month follow up in patients of both group. A. ALS-FRS and B. Forced vital capacity (FVC) in the intravenous (IV) and intrathecal (IT) groups due to worsening of patients’ conditions. ALS-FRS and FVC data are mean ± SEM.

In the IV group, 5 patients completed the 12 month follow up and 1 patient was lost to follow up after the cell transplantation. One patient, a 50-year old man with limb onset ALS, needed percutaneous endoscopic gastrostomy (PEG) placement 8 months after the cell injection and due to worsening the respiratory conditions. He underwent the tracheostomy 10 months after the injection. This patient died at the end of the study due to the respiratory infection. Other patients had decreased ALS-FRS and FVC levels during the 12 months of follow up which indicated disease progression in compare with before cell injection (Fig .3).

In the IT group, 7 patients completed the 12 month follow up and 1 patient was lost to follow up after the cell injection. One patient, a 39-year old man with limb onset ALS needed PEG placement and a tracheostomy due to worsening of his bulbar symptoms. We observed worsening of ALS and FVC percentages during 12 months of follow up in the other patients of this group (Fig .3). As presented in Figure 3, for ALS-FRS, repeated-measures ANOVA indicated a significant time effect (P<0.001) but no significant treatment effect (P=0.269). For FVC, the results of repeated-measures ANOVA also showed a significant time effect (P<0.001) but no significant treatment effect (P=0.731). Table S1 and Figure S1 summarizes additional ALS-FRS and FVC information for the study groups (See Supplementary Online Information at www.celljournal.org). 

## Discussion

We designed the present study to confirm the safety and feasibility of IV and IT transplantations of BM-MSCs in patients with ALS and compare the effects of each route of cell injection on prevention of disease progression. At the end of follow-up period we observed no local or systemic adverse effects or immediate reactions according to the clinical and laboratory assessments. One patient from the IV group experienced hypotension during the cell suspension infusion and 2 patients from the IT group complained of headaches and nausea following the IT injection. Symptoms resolved in all of these patients after treatment. MRI scans did not show any new abnormal findings such as mass formations in the brain or spinal cord. These results confirmed the safety of either cell type or routes of administration. Numerous studies have demonstrated the safety of BM-derived MSCs, which were similar to the current study (9, 15, 34, 35), but the disputable case was the transplantation pathway. Different methods of tracing in animals have shown that MSCs migrate after an IV injection and can be attracted to the damaged areas (36). The IT pathway is a direct route to reach the cerebrospinal fluid (CSF), thus the dynamic flow of the CSF helps the cells to circulate simply through the brain and spinal cord, and access impaired areas (9, 20, 37, 38) . According to these mechanisms, we expected that MSCs could improve or at least slow down the rate of disease progression, but both groups had reduced ALSFRS and FVC with disease progression during 12 months of follow up. 

The exception was patient 9 that the ALS-FRS and FVC did not change after cell therapy in comparison with before. It shows that in this patient the disease progression had a same process from 6 months before cell transplantation till 12 months after. . We could not clarified that this stability of disease process was related to stem cell activity or it was the same process as before cell transplantation.

These findings suggested that ALS probably negatively impacted BM-MSCs and reduced their quality. In order to support this hypothesis, studies have shown which ALS influenced BM-MSCs and reduced their capabilities. Secretion of trophic factors such as Insulin like growth factor-1 (IGF-1), TGF-ß, Fibroblast growth factor (FGF-2), placental growth factor (PIGF), hepatocyte growth factor (HGF), vascular endothelial growth factor (VEGF), and stromal cell derived factor 1 (SDF-1a) decreased, which correlated with progression and poor disease prognosis (39, 40). To verify our hypothesis, we intend to design a new study that investigates the effects of allogeneic stem cells obtained from healthy donors in order to locate an effective route of cell transplantation in patients with ALS. 

## Conclusion

Taken together, these results of our study demonstrated that the IV and IT injections of autologous MSCs are safe and feasible. To show the therapeutic effect of these approaches, we should perform additional clinical trials with more patients. 

## Supplementary PDF


