# Supplementary material for: Safety, Feasibility of Intravenous and Intrathecal Injection of Autologous Bone Marrow Derived Mesenchymal Stromal Cells in Patients with Amyotrophic Lateral Sclerosis: An Open Label Phase I Clinical Trial
Source: Cell J. 2018 Aug 7;20(4):592–8. doi: 10.22074/cellj.2019.5370 (PMC6099146; doi:10.22074/cellj.2019.5370)
Supplement: Supplementary file 1 [file Cell-J-20-592-s01.pdf]

www.celljournal.org

Supplementary Information for

Safety, Feasibility of Intravenous and Intrathecal Injection of Autologous Bone Marrow Derived Mesenchymal Stromal Cells in Patients with Amyotrophic Lateral Sclerosis: An Open Label Phase I Clinical Trial

Seyed Massood Nabavi, M.D.[1#], Leila Arab, M.D.[2#], Neda Jarooghi, M.Sc.[1], Tina Bolurieh, M.Sc.[1], Fatemeh Abbasi, M.Sc.[1], Soura Mardpour, Ph.D.[1], Vajihe Azimyian, Ph.D.[1], Fatemeh Moeininia, M.D.[1], Saman Maroufizadeh, M.Sc.[3], Leila Sanjari, M.D.[4], Seyedeh Esmat Hosseini, M.Sc.[5], Nasser Aghdami, M.D., Ph.D.[1*]

1. Department of Regenerative Medicine, Cell Science Research Center, Royan Institute for Stem Cell Biology and Technology, ACECR, Tehran, Iran
2. Department of Neuroscience, School of Advanced Technologies in Medicine, Tehran University of Medical Science, Tehran, Iran
3. Department of Epidemiology and Reproductive Health, Reproductive Epidemiology Research Center, Royan Institute for Reproductive Medicine, ACECR, Tehran, Iran
4. Intensive Care Unit, Mostafa Khomeini Hospital, Tehran, Iran
5. Student Research Committee, School of Nursing and Midwifery, Shahid Beheshti University of Medical Sciences, Tehran, Iran

#The first two authors equally contributed to this work.

**Corresponding Address: P.O.Box: 16635-148, Department of Regenerative Medicine, Cell Science Research Center, Royan Institute for Stem Cell Biology and Technology, ACECR, Tehran, Iran*
Email: Nasser.aghdami@royaninstitute.org

Table S1: ALS-FRS and FVC of patients in the intravenous (IV) and intrathecal (IT) groups

Patient	ALS-FRS							
	m -6	m -4	m -2	0	m 2	m 4	m 6	m 12
1	29	27	26	26	22	20	12	3
2	36	35	34	34	34	30	25	13
3	30	28	27	27	23	20	9	7
4	38	36	34	34	35	31	29	26
5	34	31	30	28	29	22	18	12
7	35	34	32	30	26	24	23	5
8	34	33	33	32	32	32	31	28
9	39	39	39	38	38	38	39	39
10	36	36	35	34	34	34	34	33
11	32	32	30	28	28	24	22	20
12	35	35	32	32	26	25	22	21
13	32	30	26	24	17	14	8	8
	**FVC**							
1	87	80	76	72	67	33.6	22	D
2	92	90	88	82	77	63	34	D
3	89	86	80	78	69	60	33	D
4	98	98	96	95	88	88	78	54
5	94	94	91	88	70	59	45	D
7	96	96	93	93	79	68	31	D
8	87	87	85	82	80	80	78	70
9	98	98	98	95	99	95	90	90
10	86	83	81	79	84	77.2	73.3	53
11	68	68	66	59.4	47	27.3	29.1	33
12	87	87	85	83	75	69	62	58
13	65	60	56	45.2	35.5	D	D	D

M; Month, ALS-FRS; ALS Functional Rating Scale, FVC; Forced vital capacity, and D; Disabled.

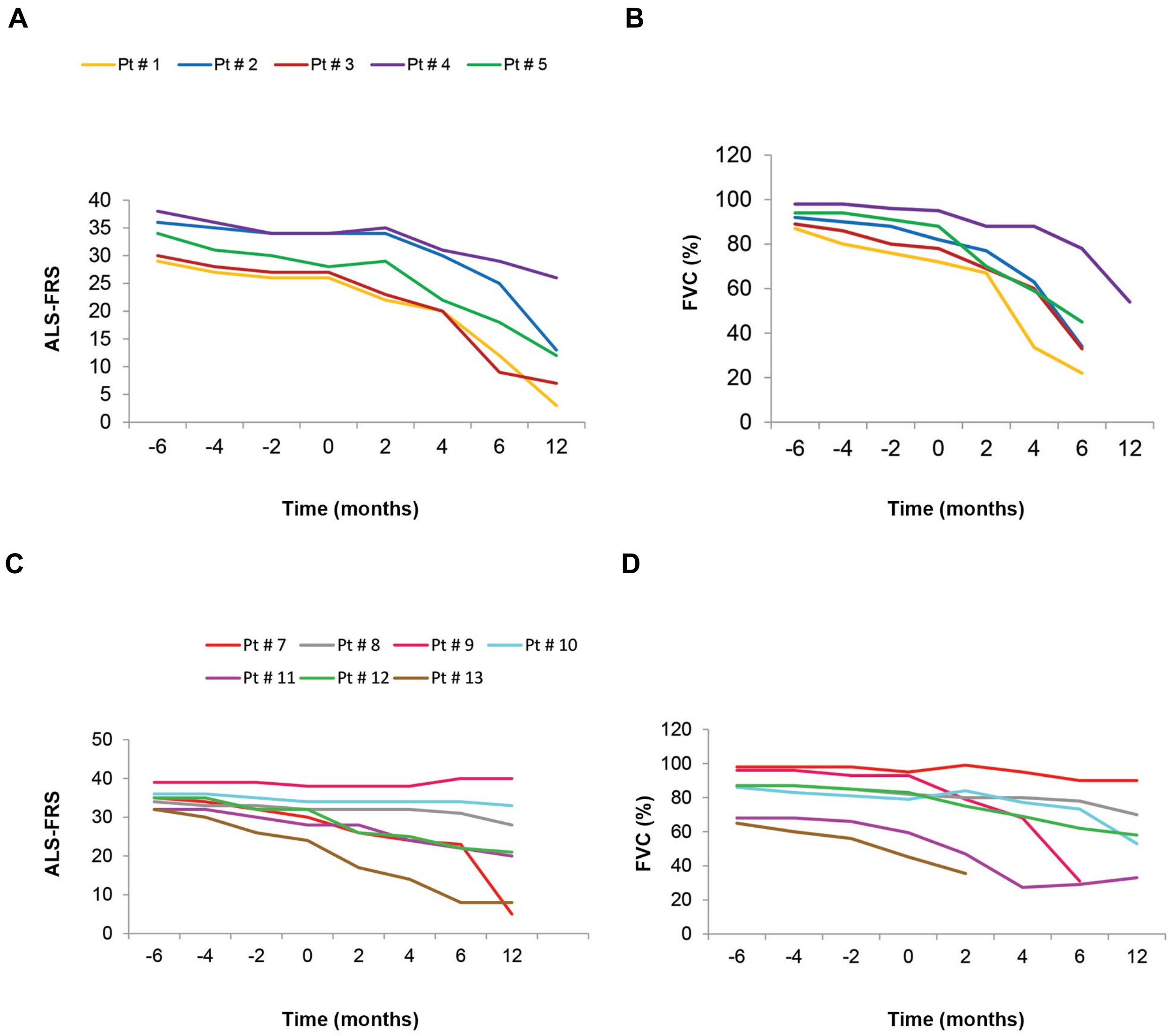

Fig.S1: The trend of ALS-FRS and FVC during 12 month follow up in patients of both group. **A.** ALS-FRS in intravenous (IV) group, **C.** ALS-FRS in intrathecal (IT) group. FVC percentages in **B.** The IV group, and **D.** IT group. In patient 13, the FVC percentage was not measurable due to respiratory insufficiency 2 months after cell transplantation.
